# A Capacitively Coupled OOK Transmitter with 4.43 dB Simulated Carrier Spectrum Peak Reduction and Enhanced CMTI for Wide-Bandgap FET Gate Driver

**DOI:** 10.3390/mi17091022

**Published:** 2026-08-28

**Authors:** Ningye He, Jiahao Li, Yongbin Huang, Jiucun Lu, Ruifang Tie, Danping Yang, Ming Yang, Zeyan Wu, Fuwei Shen, Zhenhai Chen, Zongguang Yu

**Affiliations:** 1 Engineering Technology Research Center of Intelligent Microsystems AnHui Province, Huangshan University, Huangshan 245021, China; 600103@hsu.edu.cn (R.T.); 600149@hsu.edu.cn (D.Y.); 18726206229@163.com (M.Y.); 600150@hsu.edu.cn (Z.W.); 600033@hsu.edu.cn (F.S.); 2 College of Intelligent Robotics and Advanced Manufacturing, Fudan University, Shanghai 200433, China; 21110860037@m.fudan.edu.cn (J.L.); 22110860031@m.fudan.edu.cn (Y.H.); jerrylu126@126.com (J.L.); 3 No. 58 Research Institute, China Electronic Technology Group Corporation, Wuxi 214035, China; yuzg58@163.com

**Keywords:** CMTI, EMI, modulation circuit, capacitive isolation, gate driver, common-source amplifier

## Abstract

This paper addresses the trade-off between electromagnetic interference (EMI) and common-mode transient immunity (CMTI) performance in capacitively isolated gate drivers for high-frequency silicon carbide (SiC) applications. Starting from the fundamental architecture of on-off keying (OOK) modulation, a novel transmitter (TX) modulator architecture is proposed. The proposed architecture employs a dynamic high-frequency carrier regulation circuit to suppress electromagnetic interference caused by the fixed carrier at the spectral source, while simultaneously utilizing a dynamic substrate regulation circuit to mitigate the impact of common-mode transient interference on the TX side, thereby enhancing the overall CMTI performance. The chip is fabricated in a 0.18-μm BCD process. Simulated carrier spectrum peak attenuation across different process-temperature corners demonstrates that the attenuation ranges from 4.43 dB to 19.621 dB across the fundamental through the third harmonic components. Under the same typical process-temperature corner, the simulated CMTI reaches 220 V/ns, which fully validates the effectiveness of the proposed architecture in both spectral peak reduction for EMI suppression and CMTI enhancement. Post-fabrication chip testing primarily focuses on output performance, with both the simulated carrier spectrum results for EMI and the CMTI being simulation-verified indicators. The measured output voltage range is 15–24 V; at a supply voltage of 15 V, the charging and discharging currents are 5 A and 4.5 A, respectively, and at 24 V, they are 5.7 A and 5.3 A, respectively.

## 1. Introduction

With the large-scale adoption of third-generation semiconductor SiC devices in applications such as electric vehicle (EV) traction inverters and photovoltaic (PV) energy storage systems, power device switching speeds continue to escalate, and the voltage slew rate (dV/dt) at phase-leg nodes has commonly reached the 50–150 kV/μs range [1,2,3,4,5,6,7,8,9,10]. As the sole signal transmission interface between low-voltage and high-voltage domains, capacitively isolated gate drivers are responsible for reliably transferring low-side control signals to high-side power devices, while facing the inherent trade-off between mitigating electromagnetic interference (EMI) emissions and achieving high common-mode transient immunity (CMTI).

Various optimization techniques have been proposed to address CMTI and EMI separately. For CMTI enhancement, existing solutions based on the capacitive isolation on-off keying (OOK) architecture mainly rely on receiver (RX) side front-end hardening, including high-hysteresis comparators, glitch filtering circuits, and balanced differential receiver structures, to suppress common-mode transients coupled from the high-dV/dt switching node to the RX through isolation capacitors [11,12,13,14,15,16,17,18,19,20,21,22,23,24,25,26,27,28,29]. These approaches require no modification to the core modulation architecture, and their process compatibility and reliability have been thoroughly validated in industrial scenarios. However, their noise suppression units introduce additional propagation delay and jitter, which become particularly prominent in silicon carbide (SiC) switching scenarios on the sub-100-ns time scale. Moreover, they act as passive protection mechanisms that can only filter coupled common-mode transients at the RX side, failing to reduce the common-mode coupling strength from the transmitter (TX) side modulation source, thus making it difficult to overcome the inherent trade-off between CMTI and signal transmission integrity. For EMI mitigation, existing optimizations focus on two stages: secondary-side output-stage drive and PCB-level compensation. The former softens switching edges through slew rate control, adjustable gate resistors, and Miller clamping to reduce broadband radiation, but suffers from an inherent trade-off between switching speed and power loss that is difficult to reconcile in high-frequency SiC scenarios. The latter reduces the common-mode loop area through symmetric isolation capacitor layout, ground plane optimization, and high-frequency decoupling, acting only as a board-level remedy that does not address the OOK carrier itself [30,31,32,33,34,35,36,37,38,39,40,41,42]. In summary, existing solutions lack architecture-level synergy in optimizing CMTI and EMI, making it difficult to overcome multiple performance trade-offs.

To address the above issues, this paper proposes a TX-side collaborative optimization strategy that originates from the OOK modulation architecture. This strategy suppresses EMI radiation at the spectral origin while enhancing CMTI immunity, thereby breaking the performance trade-off inherent in conventional separate optimization schemes. It should be noted that the TX-side CMTI enhancement scheme and the test chip employed in this work are based on the same chip and fabrication batch as those reported in [28,29]. The main novelty of this work lies in the dynamic high-frequency carrier regulation module proposed for EMI radiation suppression. Based on this architecture, the designed chip is evaluated through simulation under the typical process-temperature corner. The simulation results show a worst-case spectral peak attenuation of 4.43 dB and a CMTI of 220 V/ns.

## 2. System Architecture and Analysis

### 2.1. System Architecture

**Figure 1:**
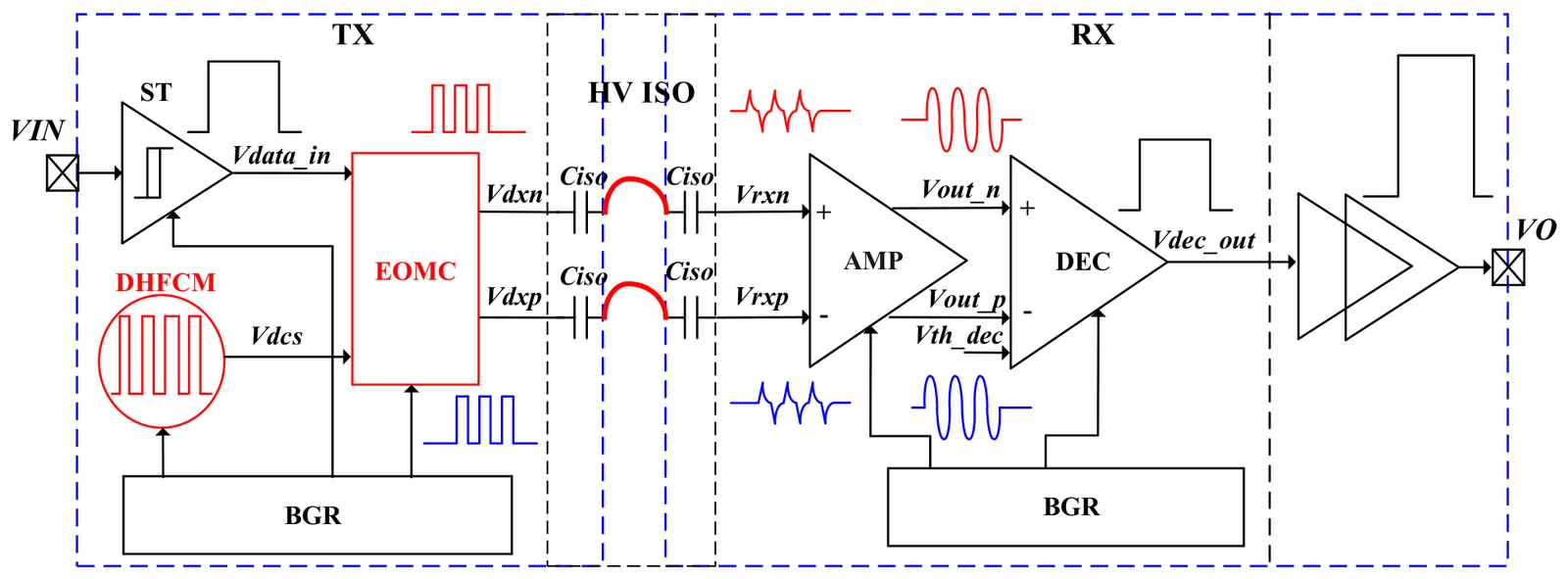
System architecture of a high-CMTI low-EMI capacitively isolated gate driver chip.

The proposed capacitively isolated gate driver architecture in this paper is shown in Figure 1, which adopts a TX/RX dual-chip design with high-voltage isolation capacitors Ciso integrated on both sides to achieve cross-barrier transmission and high low-voltage isolation. On the TX side, the input signal VIN is shaped by a Schmitt trigger (ST) to obtain Vdata_in. A dynamic high-frequency carrier module (DHFCM) generates a time-varying spread-spectrum signal Vdcs to suppress narrowband EMI. These two signals are fed into an enhanced OOK modulation circuit (EOMC), which outputs complementary differential signals Vdxn and Vdxp. This pair of signals is coupled to the secondary side through the high-voltage isolation capacitor (HV ISO) Ciso, serving both as the modulated signal for cross-barrier transmission and as the primary path through which high dV/dt common mode noise couples to the secondary side. The proposed architecture reduces common mode coupling efficiency through dynamic common mode substrate modulation and is complemented by a CMTI event-triggered common mode current discharge path. It actively suppresses interference from the TX side, thereby overcoming the speed-immunity trade-off without relying on high-hysteresis filtering on the RX side, and significantly improves CMTI immunity. On the RX side, the coupled weak differential signals Vrxn and Vrxp are first differentially amplified to obtain the amplified signals Vout_n and Vout_p. Subsequently, a decoder (DEC) performs a logic decision on Vout_n and Vout_p based on the threshold voltage Vth_dec and decodes them into the corresponding digital signal Vdec_out. Finally, Vdec_out is power-amplified by the driver stage to generate the power drive signal VO.

### 2.2. Analysis of CMTI

**Figure 2:**
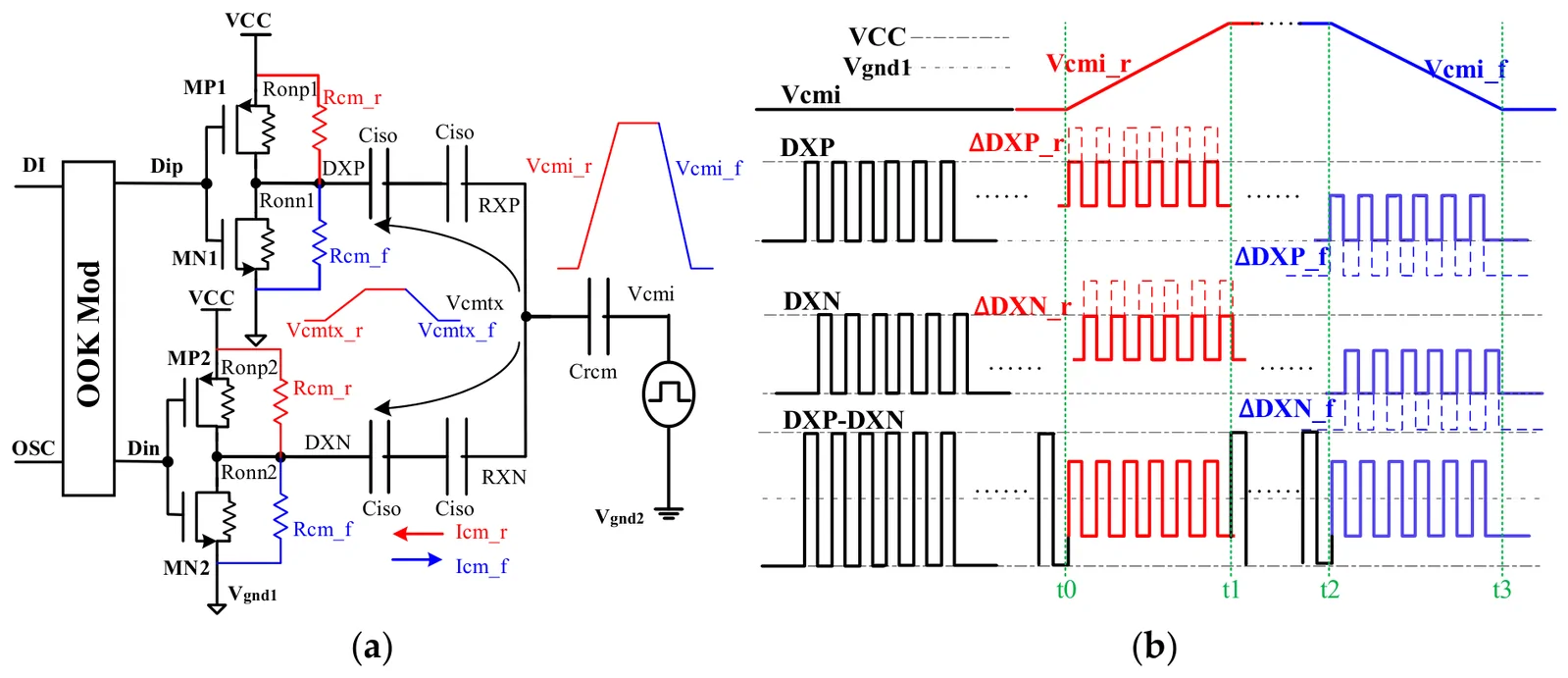
Circuit and waveform diagrams for analysis of CMTI interference on the TX side. (**a**) Circuit schematic. (**b**) Key operating waveforms.

For capacitively isolated gate drivers, the TX circuit and the RX circuit are connected to the primary-side ground Vgnd1 and the secondary-side floating ground Vgnd2, respectively. Electrical isolation between the two is achieved by high-voltage isolation capacitors. However, in applications such as half-bridge configurations, the high-side Vgnd2 floats significantly with respect to Vgnd1. The high dV/dt transients between the two grounds couple through the isolation capacitors and generate common-mode transient interference. This interference becomes a primary threat to system reliability. To analyze the impact of common-mode transient interference on the TX side in depth, Figure 2a presents a simplified circuit schematic of the TX side subject to Common-Mode Transient (CMT) interference. In this figure, Rcm_r and Rcm_f are equivalent common-mode impedances. They are introduced to quantify the common-mode coupling efficiency under high dV/dt. The TX-side output stage consists of two inverter pairs MP1/MN1 and MP2/MN2. Their on-resistances are Ronp and Ronn. Crcm is the common-mode equivalent capacitance at the RX side.

Figure 2b shows the key operating waveform diagram illustrating the impact of common-mode interference on the circuit. Before t0, Vgnd2 experiences no common-mode transient, and Vcmi is zero. When DI is high, the complementary single-ended signals DXP and DXN have a high level of VCC and a low level of GND. Their differential output, DXP−DXN, has a high level of VCC and a low level of −VCC. Overall, the circuit remains in a steady state without distortion. During the interval t0–t1, the common-mode transient Vcmi_r of Vgnd2 rises linearly. This transient generates a common-mode interference voltage Vcmtx through coupling via the capacitor Crcm. Simultaneously, it injects common-mode interference currents Icm_r into the DXN and DXP nodes through the high-voltage isolation capacitor Ciso. As a result, the output levels at DXN and DXP are elevated by ΔDXN_r and ΔDXP_r. This interference can be equivalently modeled by introducing an equivalent common-mode impedance Rcm_r across the source–drain terminals of MP1 and MP2. When MP is on, Ronp is in parallel with Rcm_r, which accelerates the pull-up of the DXN and DXP nodes to VCC. When MN is on, Ronn is in series with Rcm_r, raising the output levels of DXN and DXP. Based on this, Rcm_r can be used to quantitatively model the common-mode interference during the t0–t1 period. During the interval t1–t2, the common-mode transient Vcmi of Vgnd2 remains stable without any voltage change. Therefore, the high-voltage isolation capacitor Ciso no longer induces common-mode interference currents. The level elevation effects on DXP and DXN disappear. The outputs return to their steady-state values of high level VCC and low level GND. During the interval t2–t3, the common-mode transient Vcmi_f of Vgnd2 decreases linearly. This decreasing transient generates common-mode interference currents Icm_f flowing out of the DXN and DXP nodes through the capacitor Ciso. Consequently, the output signal amplitudes at DXN and DXP are reduced by ΔDXN_f and ΔDXP_f, respectively. This interference can be equivalently modeled by introducing an equivalent common-mode impedance Rcm_f across the source–drain terminals of MN1 and MN2. When MP is on, Ronp is in series with Rcm_f, thereby lowering the output levels of DXN and DXP. When MN is on, Ronn is in parallel with Rcm_f, accelerating the pull-down of the DXN and DXP nodes to GND. Based on this, Rcm_f can be used to quantitatively model the common-mode interference during the t2–t3 period.

On the TX side, the common-mode transient voltage Vcmi on the floating ground Vgnd2 induces a common-mode interference voltage Vcmtx at the TX side. This reduces the effective swing of the differential output signal DXP − DXN. If the amplitude of Vcmtx exceeds a certain threshold, the effective swing of the differential signal drops below the recognition lower limit. Consequently, the RX side fails to correctly decode the OOK modulation logic. This eventually leads to decoding mis-triggering.

### 2.3. Analysis of EMI

**Figure 3:**
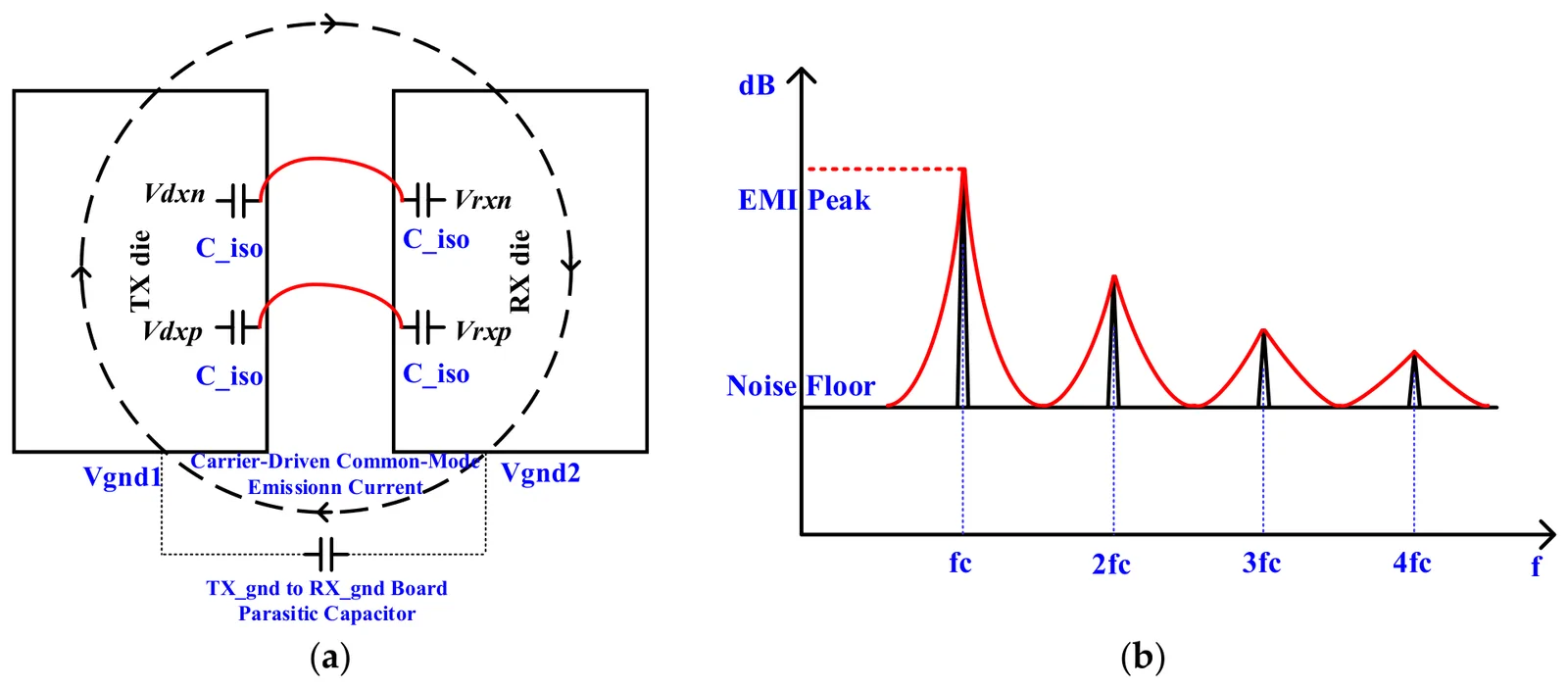
Analysis of EMI interference sources in the OOK architecture. (**a**) Circuit schematic. (**b**) Frequency spectrum.

For capacitively isolated gate drivers, there are multiple EMI sources, such as secondary-side switching noise and power supply oscillations. However, the factor that most constrains electromagnetic compatibility (EMC) compliance is the periodic charging and discharging of the cross-barrier high-voltage isolation capacitor Ciso under the OOK architecture. As shown in Figure 3a, the TX side outputs complementary high-frequency signals Vdxn/Vdxp to charge and discharge Ciso. During this charging and discharging, the primary side and secondary side grounds (GND1 and GND2) are completely floating and have no low-impedance return path. Consequently, the current is forced to flow through the interlayer parasitic capacitances of the PCB, forming a large common mode current loop. This loop acts as an efficient radiating antenna. It is a narrowband radiation source specific to the capacitive isolation architecture. This source directly governs the EMI performance of the system. Therefore, it also serves as the primary focus for the subsequent dynamic spread-spectrum optimization.

This physical mechanism manifests in the frequency domain as a typical narrowband discrete spectrum. As shown in Figure 3b, the energy of the fixed carrier is highly concentrated at the fundamental frequency fc and its harmonic components. This results in sharp interference peaks. These peaks are highly likely to overlap with mainstream wireless communication bands, such as 2.4 GHz Wi Fi and 5G. Although the noise floor is very low, the excessively high single-frequency peak values make conventional shielding and filtering methods ineffective. This exposes the inherent challenge faced by the fixed frequency OOK architecture in meeting general EMC requirements.

The above analysis indicates that the EMI problem of the conventional fixed carrier scheme is not caused by excessive total radiated energy. Instead, it arises from the excessive concentration of energy in the frequency domain. This “spike-like” spectral distribution characteristic implies that peripheral circuit optimization alone cannot fundamentally eliminate the risk of exceeding peak limits. Therefore, it is necessary to address the issue at the modulation architecture level by modifying the spectral energy distribution of the carrier to suppress narrowband radiation.

## 3. Circuit Implementation

### 3.1. Dynamic High-Frequency Carrier Module

**Figure 4:**
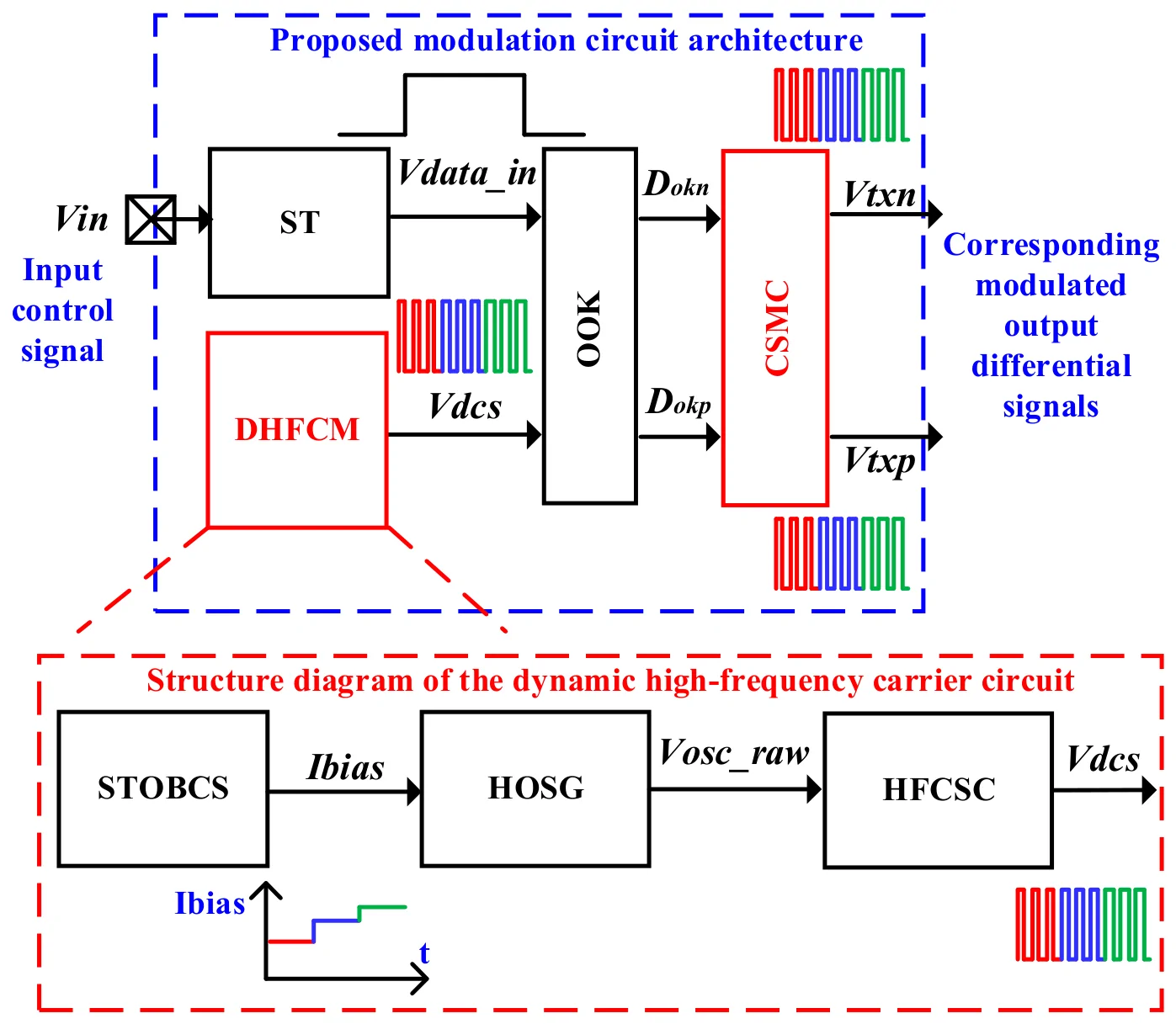
Architecture diagram of DHFCM.

Figure 4 illustrates the architecture diagram of the proposed DHFCM. The circuit module is composed of a self-tuned oscillating bias current source (STOBCS), a high-frequency oscillation signal generation circuit (HOSG), and a high-frequency clock shaping circuit (HFCSC). For EMI suppression, the DHFCM employs the STOBCS to generate a time-varying current Ibias. This current drives the HOSG to output a raw signal Vosc_raw, which is related to Ibias. After being shaped by the HFCSC, a high-frequency carrier Vdcs with dynamically time-varying frequency is finally generated. Consequently, the concentrated energy is dispersed over a wider bandwidth, leading to a significant reduction in radiated interference.

**Figure 5:**
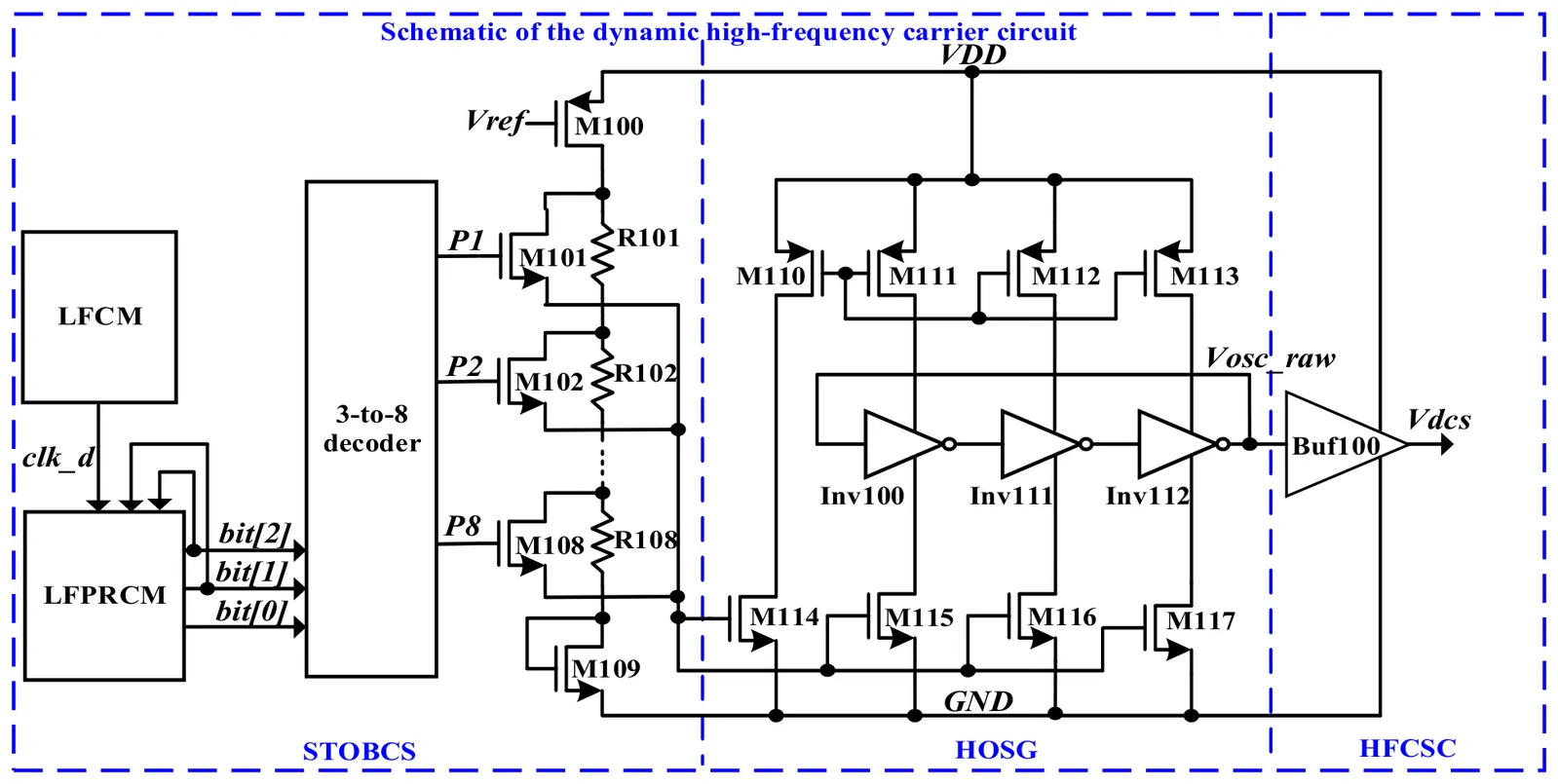
Schematic of DHFCM.

Figure 5 shows the schematic of the proposed DHFCM. It employs a pseudo-random frequency modulation scheme to spread the spectral energy, serving as the core unit for architecture-level EMI optimization. The low-frequency clock module (LFCM) generates the low-frequency clock signal clk_d, which serves as the driving clock for the low-frequency pseudo-random control module (LFPRCM). Within the LFPRCM, a logic control unit produces a dynamically varying 3-bit control signal bit[2:0]. This signal is decoded by a 3-to-8 decoder to output eight interleaved selection signals P1-P8, which precisely regulate the output current Ibias of the self-tuned bias current source. The time-varying bias current is injected into the core of a three-stage ring oscillator, directly modulating the instantaneous frequency of the raw high-frequency signal Vosc_raw, causing it to dynamically jitter with Ibias. After processing by a high-frequency shaping circuit, the circuit finally produces a stable dynamic spread-spectrum carrier, Vdcs. This pseudo-random frequency modulation mechanism flattens the highly concentrated energy of the fixed carrier over a wide frequency band, significantly reducing the power spectral density at individual frequencies. Thus, it solves the EMI over-limit issue of conventional fixed carriers at the modulation source.

**Figure 6:**
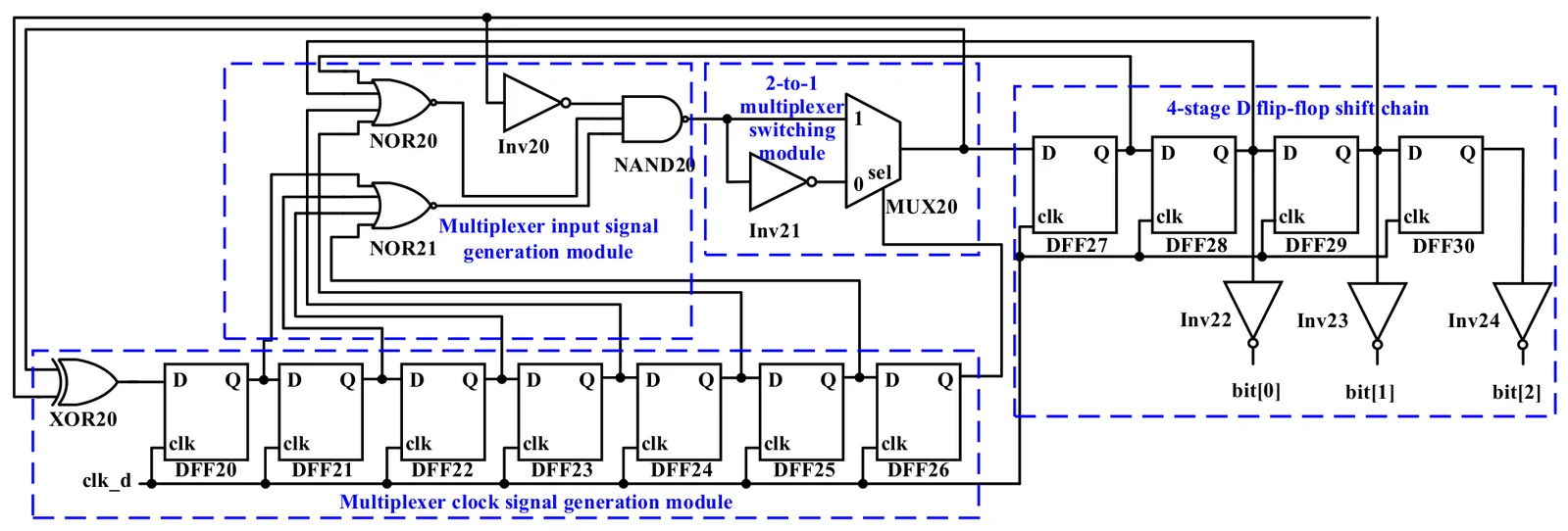
Schematic of the LFPRCM.

Figure 6 presents the implementation schematic of the LFPRCM. This module is based on a feedback shift register architecture. It is collaboratively constructed by a sequential shift chain, a combinational logic feedback network, a 2-to-1 multiplexer switching module, and a multiplexer clock generation module. It is used to generate a 3-bit low-frequency pseudo-random code bit[2:0]. Four cascaded D flip-flops DFF27-DFF30 form the main shift chain. After being conditioned by inverters Inv22-Inv24, the outputs of DFF28-DFF30 are respectively mapped to bit0-bit2. The output of DFF29 and the input of DFF27 are fed into XOR gate XOR20. The resulting signal serves as the multiplexer clock for the seven-stage timing chain formed by DFF20-DFF26. In addition, the outputs of DFF27-DFF29 and those of DFF20-DFF25 are combined through NOR gates NOR20 and NOR21 and inverter Inv20. The combined signal is then fed into NAND gate NAND20. The output of NAND20 serves as the feedback signal. This feedback signal is conditioned by inverter Inv21. Then, it undergoes polarity switching by the 2-to-1 multiplexer MUX20 under the control of the SEL signal. Finally, it is fed back to the input of DFF27 to form a closed-loop iteration. This design uses the XOR gate to establish cross-stage state correlation. It also dynamically adjusts the feedback polarity through MUX20, effectively breaking the periodicity of the sequence. This ensures that the output pseudo-random code possesses high random statistical characteristics to meet the timing requirements for dynamic spread-spectrum carrier modulation.

**Figure 7:**
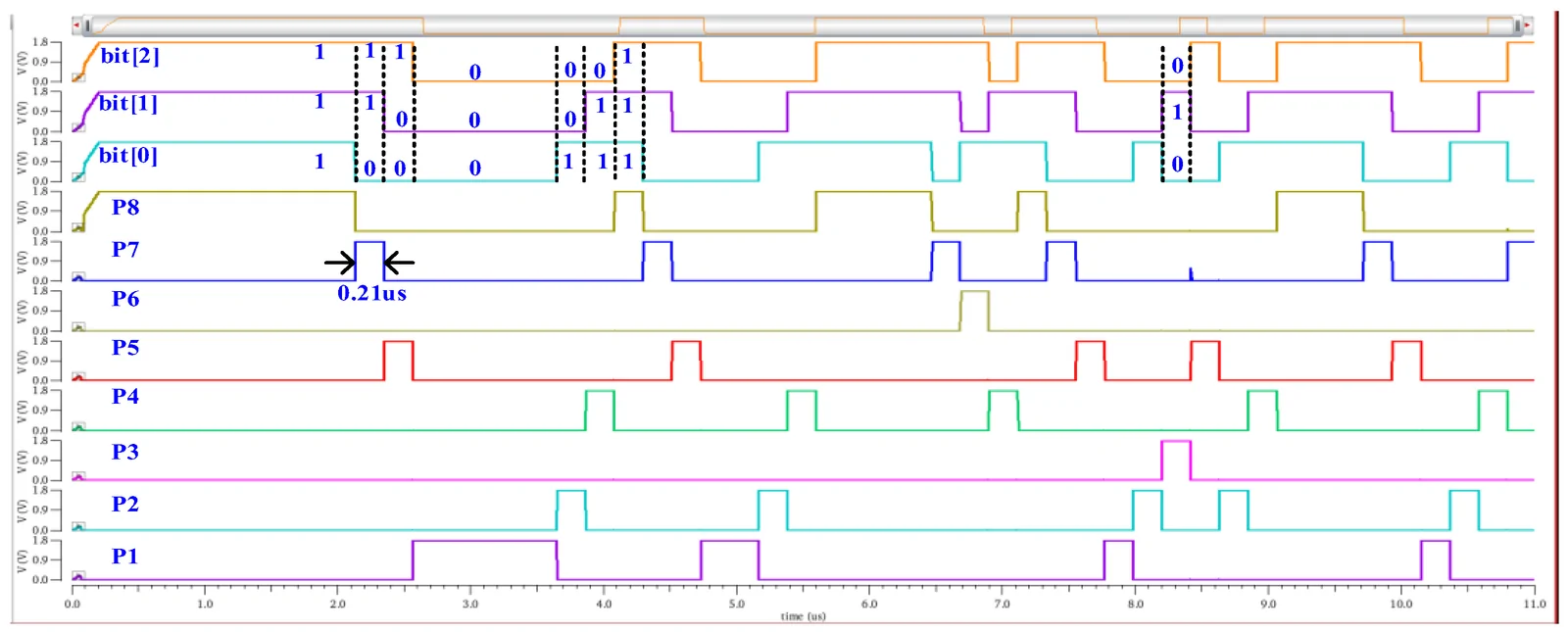
Simulation waveforms of the 3-to-8 decoder in the dynamic high-frequency carrier circuit.

Figure 7 illustrates the simulated output timing waveforms of the 3-to-8 decoder in the dynamic high-frequency carrier circuit. It is observed that the output bit [0]-bit[2] of the LFPRCM change approximately every 0.21 μs. Accordingly, the frequency of the low-frequency clock module is estimated to be approximately 4.7 MHz. In addition, exactly one of the decoder outputs P1-P8 is high for each bit[0]-bit[2] state. This ensures the one-hot selection of the current source branches. Meanwhile, the variation sequence of bit[0]-bit[2] exhibits non-periodic characteristics over short time scales, with no discernible pattern. This validates the correctness of the operation logic of the self-tuned bias current source module. Specifically, the module generates pseudo-random control signals, thereby suppressing discrete modulation sidebands in the frequency spectrum.

**Figure 8:**
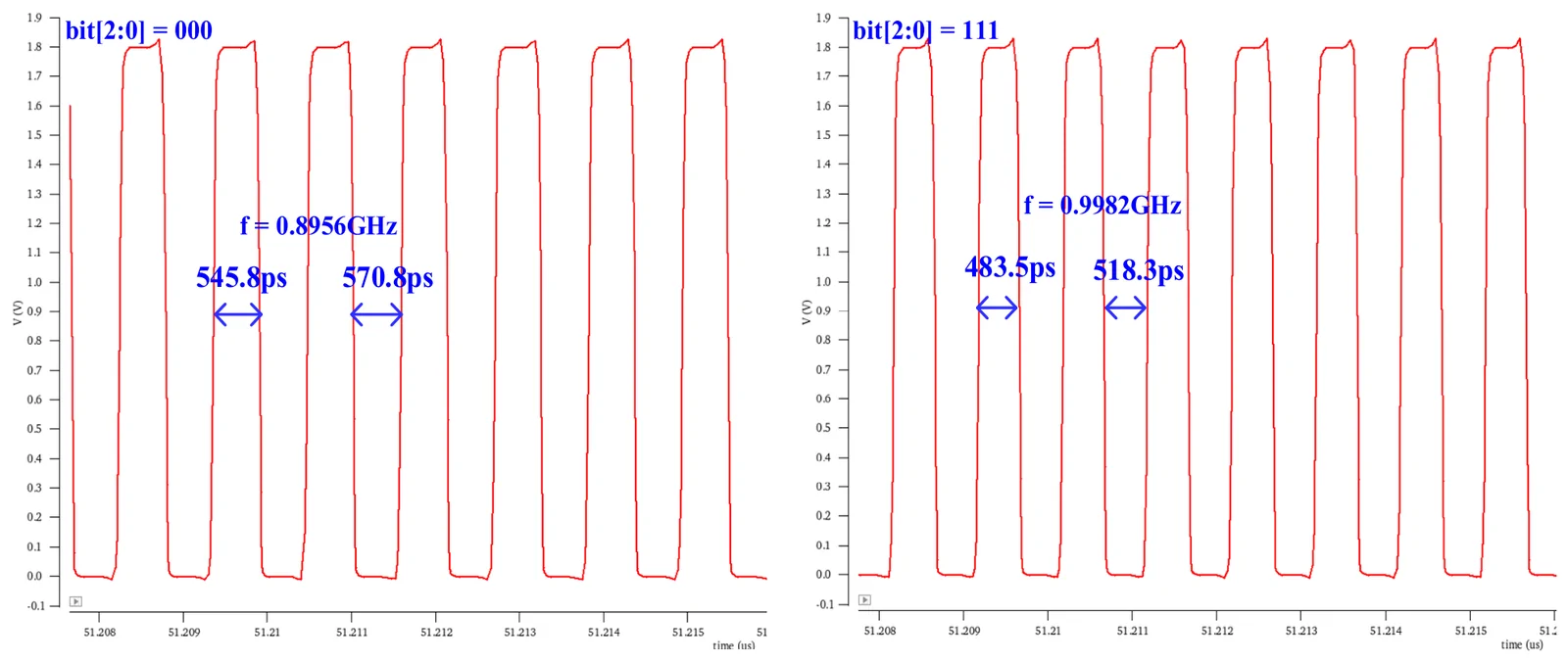
Simulation waveforms of the dynamic carrier output frequency.

Figure 8 presents the simulated waveforms of the dynamic high-frequency carrier circuit under two operating states corresponding to the minimum and maximum output frequencies. The simulation is performed under the TT process corner at 25 °C. From the waveform data, it can be seen that when the control code bit[2:0] = 000, the pull-up current provided by the current source array is approximately 80 μA, and the pull-down current is approximately 88 μA. Under this bias condition, the high-level duration of the output signal is about 545.8 ps, and the low-level duration is about 570.8 ps. Thus, the resulting single cycle length is 1.116 ns, corresponding to an oscillation frequency of approximately 895.6 MHz. When the control code is switched to bit[2:0] = 111, the pull-up current increases to about 92 μA, and the pull-down current rises to about 113 μA. In this case, the output signal period is reduced to approximately 1 ns, and the frequency correspondingly increases to 998.2 MHz. Further calculation yields a carrier center frequency fc of about 946.9 MHz, a frequency deviation Δf of 102.6 MHz, and a spreading ratio δ of 10.8%. This spreading ratio is considerably higher than that of conventional designs and is expected to provide a more significant suppression effect on EMI peaks.

To verify the robustness of the dynamic high-frequency carrier Vdcs across all process corners and a wide temperature range, corner simulations are performed at TT, SS, and FF process corners and at three temperature points of −25 °C, 25 °C, and 100 °C. The results are summarized in Table 1. At the TT corner, the output frequency exhibits a decreasing trend as temperature rises. The frequency deviation Δf increases from 89.4 MHz to 111.8 MHz, and the spreading ratio δ rises from 8.4% to 13.6%. The variation magnitude remains well controlled. At the SS slow corner, the output frequencies are generally lower. Under the worst-case condition of the SS corner at 100 °C, fmin is 625.9 MHz, fmax is 701.9 MHz, and Δf is 76.0 MHz. Although these values are lower than the nominal target frequency, this carrier is used only for OOK modulation, and the receiver employs an envelope detection architecture, which is insensitive to absolute frequency accuracy. Thus, the deviation remains within an acceptable range. At the FF fast corner, the output frequencies are generally higher. At −25 °C, fmax reaches 1.355 GHz, fmin is 1.205 GHz, Δf is 149.6 MHz, and δ is 11.7%. The spreading performance meets the requirements. Overall, the designed dynamic high-frequency carrier module maintains a sufficient frequency deviation range across the three process corners and the wide temperature range from −25 °C to 100 °C.

**Figure 9:**
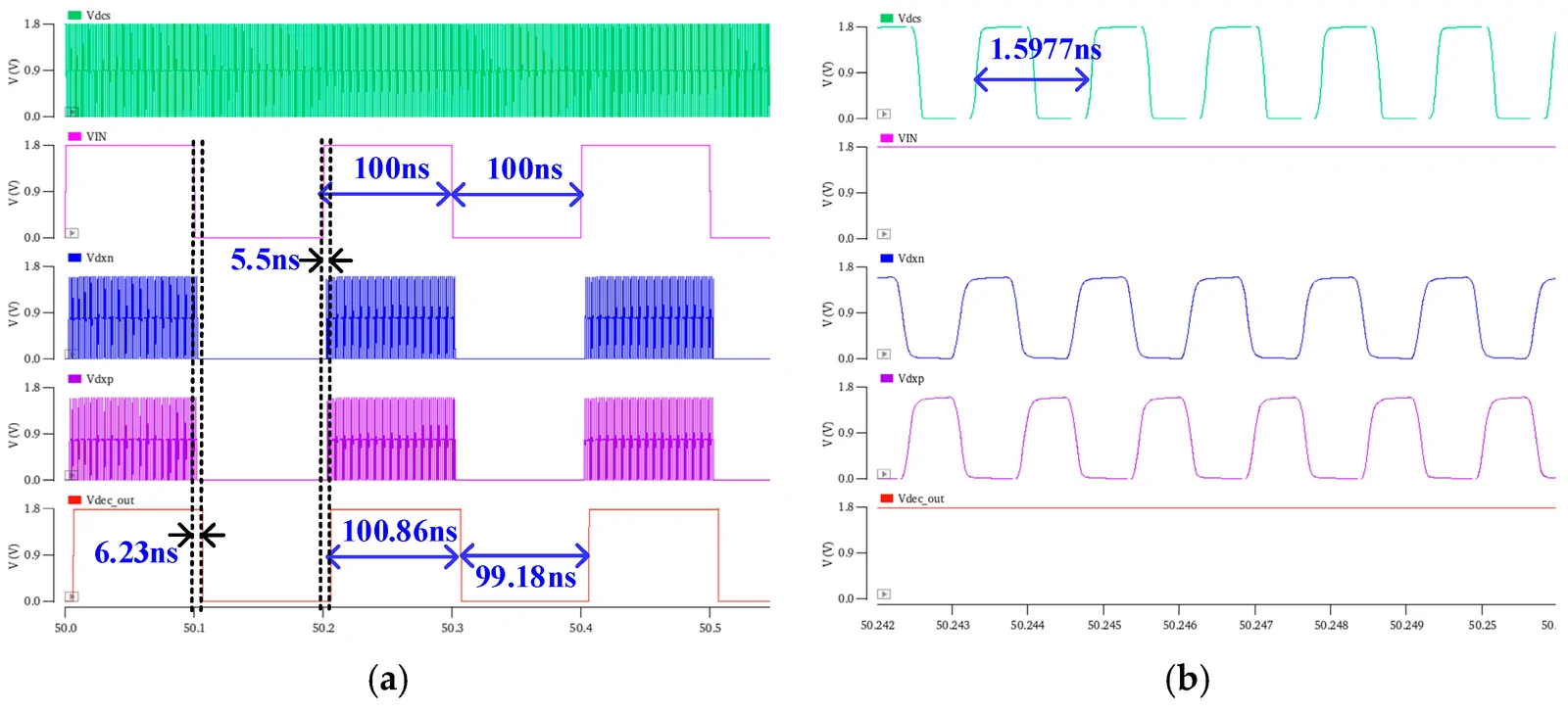
Simulated system operation waveforms of Vdcs at the minimum carrier frequency of 0.6259 GHz. (**a**) Overall waveform. (**b**) Enlarged view.

**Figure 10:**
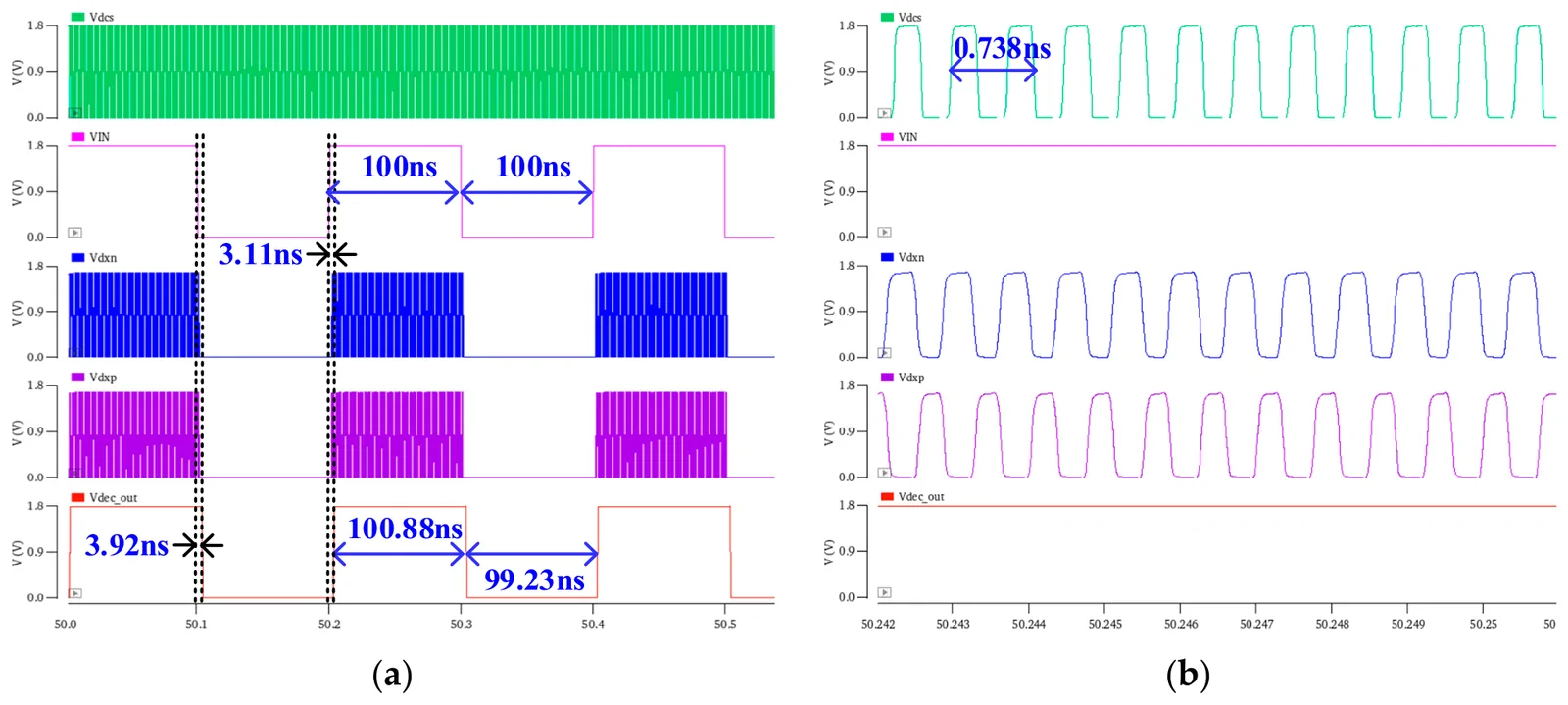
Simulated system operation waveforms of Vdcs at the maximum carrier frequency of 1.355 GHz. (**a**) Overall waveform. (**b**) Enlarged view.

To verify that the dynamic high-frequency carrier Vdcs can ensure proper system operation across different process corners and temperature conditions, this paper manually sets the control signal values at the input of the 3-to-8 decoder to fix Vdcs at its minimum and maximum frequencies, respectively, and then observes whether the system can correctly decode the transmitted control signal. Figure 9 presents the simulation results at the minimum carrier frequency of 0.6259 GHz under the SS process corner and an ambient temperature of 100 °C, with a 5 MHz high-frequency control signal applied at the transmitter side. The simulation waveforms demonstrate that the system can output the correct decoded signal Vdec_out. Under this condition, the rising-edge propagation delay from VIN to Vdec_out is 5.5 ns, and the falling-edge propagation delay is 6.23 ns. Figure 10 presents the simulation results at the maximum carrier frequency of 1.355 GHz under the FF process corner and an ambient temperature of −25 °C, with the same 5 MHz control signal. The system also successfully obtains the correct decoded signal Vdec_out, with a rising-edge propagation delay of 3.11 ns and a falling-edge propagation delay of 3.92 ns. These results indicate that the system operates correctly across the entire frequency tuning range of the dynamic high-frequency carrier Vdcs, fully verifying that the proposed module design satisfies the system requirements.

**Figure 11:**
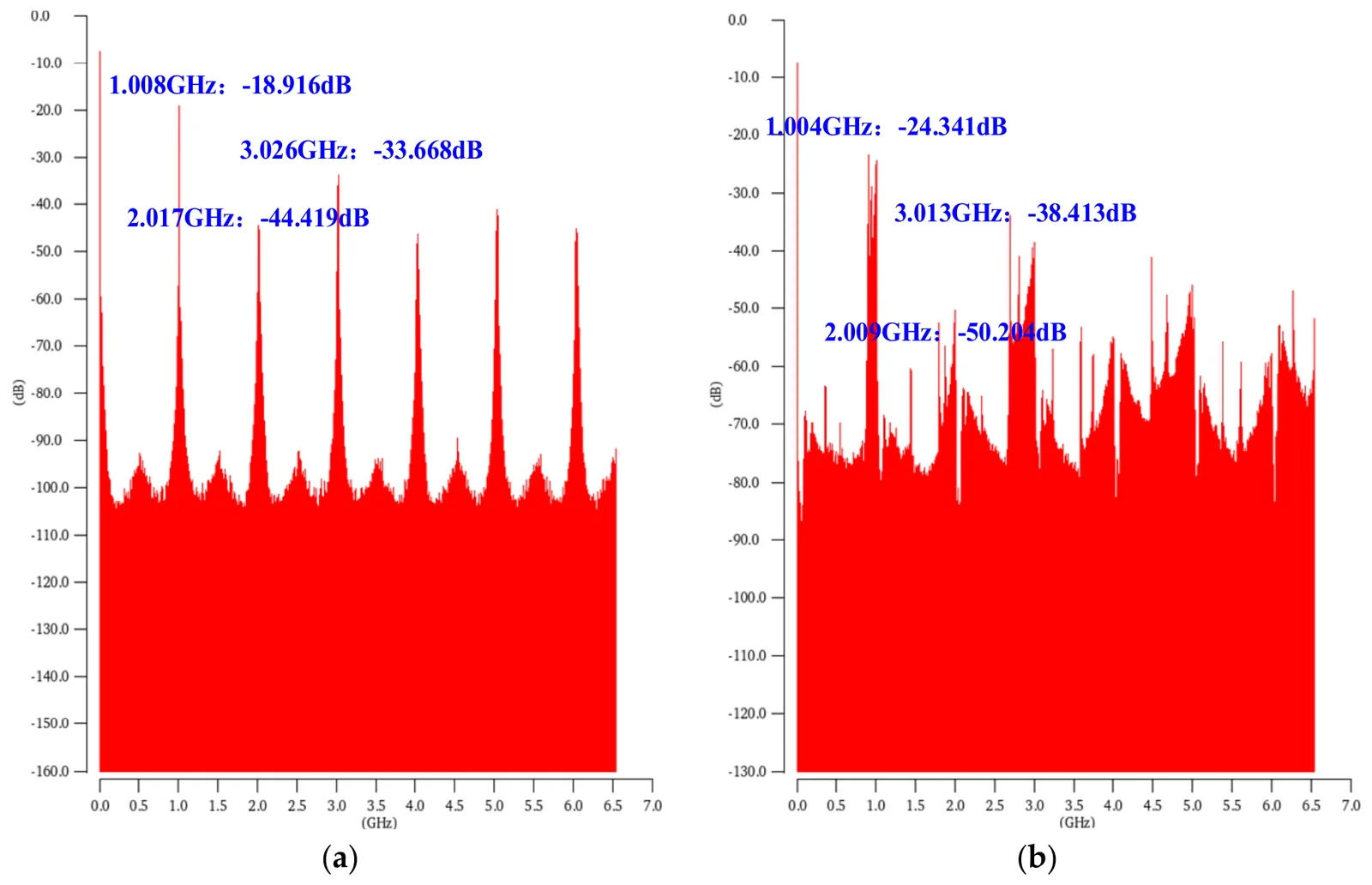
Output spectrum comparison of the carrier. (**a**) Fixed carrier. (**b**) Dynamic carrier.

Figure 11 provides a visual comparison between the output spectrum of the proposed spread-spectrum dynamic high-frequency carrier and that of a conventional single-frequency carrier without spread spectrum. For the fixed-frequency carrier, its frequency is set by fixing the input control signals of the 3-to-8 decoder, approximately at the midpoint between the minimum and maximum output frequencies of the dynamic carrier. The spectrum is calculated based on a 10 μs time-domain window from 0.5 μs to 10.5 μs, with 131,072 sampling points and an effective sampling rate of 13.11 GSa/s, corresponding to a frequency resolution of 100 kHz. The effective FFT analysis frequency range is from 100 kHz to the Nyquist frequency of 6.554 GHz, which fully covers the fundamental and third-harmonic components. A Blackman-Harris window is applied to suppress spectral leakage, and no spectral averaging is employed to preserve the detailed characteristics of the original spectrum. The spectrum vertical axis is in dBV with a reference of 1 V, and no additional peak normalization is performed, with Peak Sat. Level set to 0. The spectral display range is from 0 to 6.554 GHz, the full Nyquist frequency range, to present the spectral characteristics of the fundamental and third-harmonic components. Both the fixed carrier and the dynamic spread-spectrum carrier adopt identical FFT settings to ensure a fair EMI performance comparison. The simulation is performed under the TT process corner at 25 °C. The results show that, compared with the fixed-frequency carrier, the dynamic carrier achieves a maximum spectral peak attenuation of 5.425 dB at the fundamental frequency, 5.785 dB at the second harmonic, and 4.745 dB at the third harmonic. In addition to peak suppression, the signal energy is simultaneously dispersed over a wider frequency range, thereby effectively reducing the radiated intensity of electromagnetic interference. These simulation results fully validate the correctness and effectiveness of the proposed low-EMI dynamic high-frequency carrier circuit design.

Table 2 compares the FFT spectral peaks of the fixed carrier and the dynamic high-frequency carrier under multiple process-temperature conditions, listing the simulated amplitudes of the fundamental, second-harmonic, and third-harmonic components. Under all simulation conditions, the dynamic carrier effectively suppresses the peak levels of each harmonic component. Benefiting from the spread-spectrum effect, the maximum attenuation of the fundamental peak is 15.368 dB, occurring at the FF corner and 25 °C, while the minimum attenuation is 4.439 dB at the TT corner and −25 °C. For the second harmonic, the maximum attenuation is 18.125 dB at the TT corner and 100 °C, and the minimum is 4.571 dB at the TT corner and −25 °C. For the third harmonic, the maximum attenuation is 19.621 dB at the TT corner and 100 °C, and the minimum is 4.633 dB at the TT corner and −25 °C. Process and temperature variations affect the dithering modulation effect, resulting in a condition-dependent EMI suppression performance. Under the same condition, the maximum attenuation among the fundamental, second-harmonic, and third-harmonic components is adopted as the EMI evaluation metric. All simulations are performed with identical FFT parameter settings.

### 3.2. Enhanced OOK

**Figure 12:**
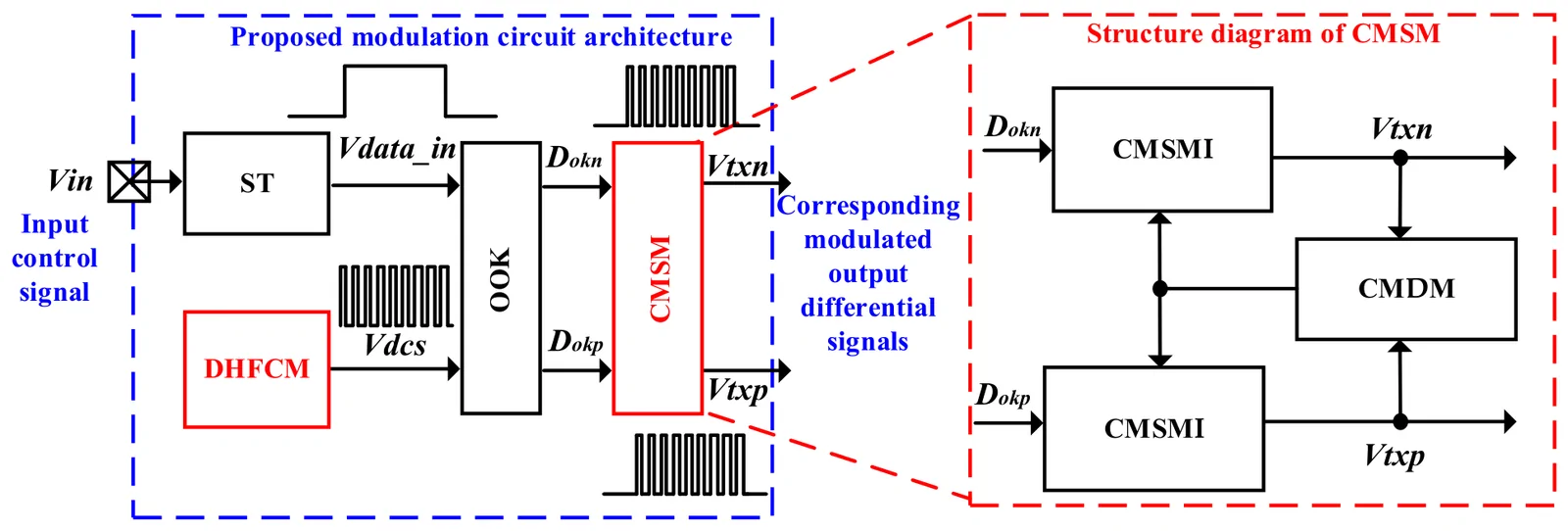
Architecture diagram of the common-mode substrate modulation circuit.

The architecture of the proposed modulation circuit is shown in Figure 12. To address CMT issues, the proposed enhanced OOK modulation circuit incorporates a common-mode substrate modulation circuit (CMSM) into the conventional OOK architecture. This CMSM consists of a common-mode substrate modulation inverter (CMSMI) and a common-mode signal detection module (CMDM). The OOK processed signals Dokn and Dokp are fed into a buffer inverter with substrate modulation, which generates the differential output signals Vtxn and Vtxp. Upon detection of common mode interference, the circuit dynamically adjusts the substrate potential of the output inverter and reconfigures the common mode current discharge path. As a result, common mode transient interference is effectively suppressed.

**Figure 13:**
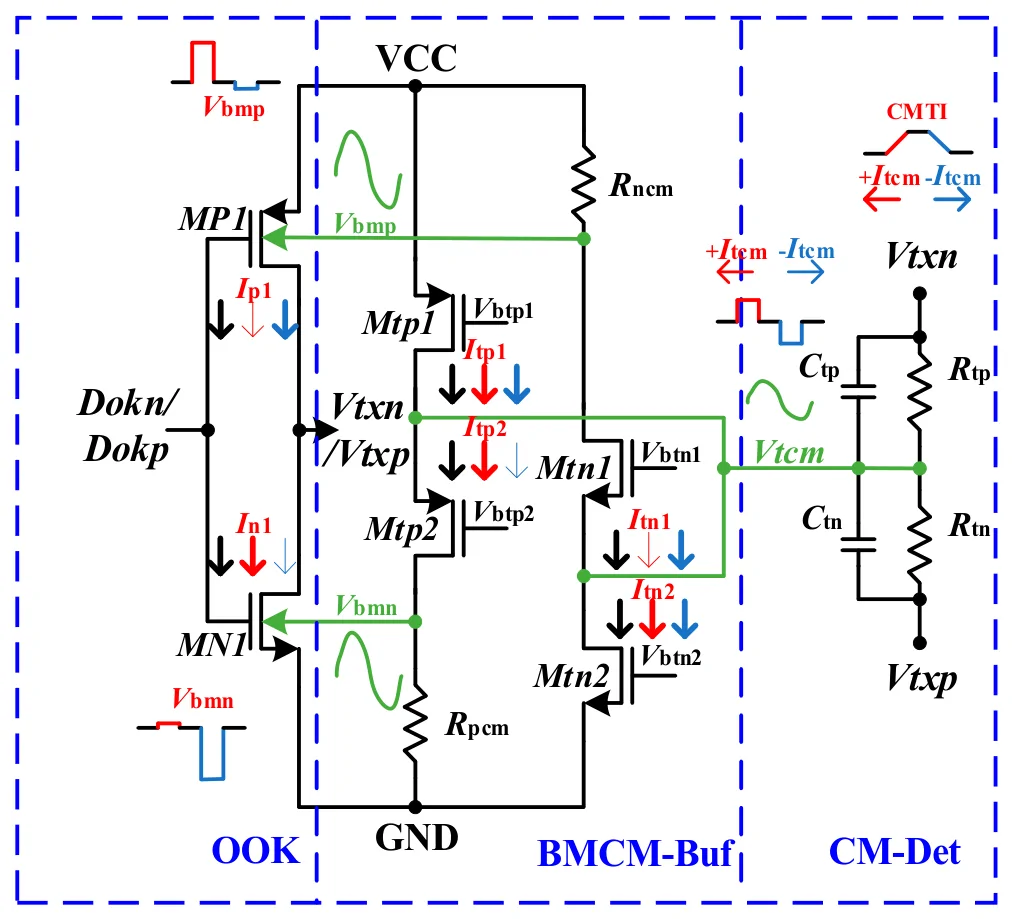
Schematic of the enhanced OOK modulation circuit.

The schematic of the proposed common mode substrate modulation circuit is shown in Figure 13. Unlike conventional OOK buffer inverters, the substrate potentials of the inverters MP1 and MN1 used in this work are no longer fixed at VCC and GND. Instead, they adopt feedback modulation voltages that are related to the output signal Vtcm of the common mode detection circuit. The common mode detection employs resistive summing detection. Capacitors Ctp and Ctn serve as filter capacitors. The detected common mode signal Vtcm is fed into the Bulk Modulated Common-Mode Buffer (BMCM-Buf) circuit. This circuit generates two substrate bias voltage signals, Vbmp and Vbmn. Consequently, the impact of CMTI events is suppressed.

When a +CMT event occurs, a current +Itcm flows into the Vtcm node. In response, the substrate potential Vbmp of MP1 in the OOK circuit rises. Meanwhile, the current Itn1 of Mtn1 in the BMCM-Buf circuit decreases. This synergistic action rapidly suppresses the interference current flowing toward the TX side chip. When a -CMT event occurs, a current -Itcm flows out of the Vtcm node. Correspondingly, the substrate potential of MN1 in the OOK circuit decreases. In this case, the current Itp2 of Mtp2 in the BMCM-Buf circuit decreases. The interference current flowing out of the TX-side chip is rapidly attenuated by this combined action. The CMTI robustness of the circuit is significantly improved by this dual-path suppression mechanism, which guarantees the validity of the output signal swing at the transmitter side during CMT events.

**Figure 14:**
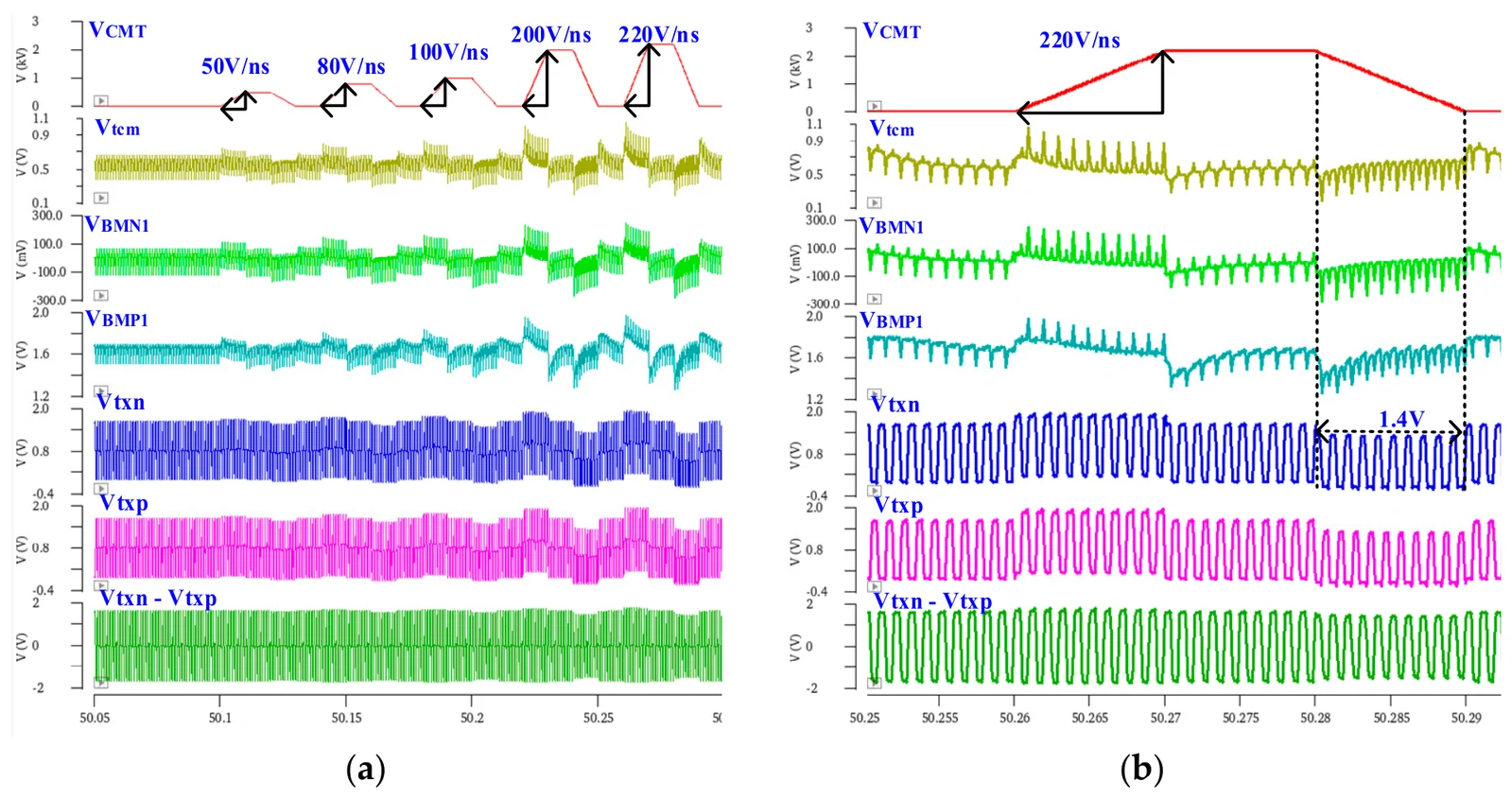
Simulation results of the enhanced OOK architecture. (**a**) Overall waveform. (**b**) Enlarged view.

Figure 14 presents the simulated waveforms of the key nodes in the proposed enhanced OOK modulation circuit under a CMTI event. In the simulation, a common-mode interference signal is injected between the TX and RX ground terminals to examine the response characteristics of the key nodes in the OOK modulator and the differential outputs TXN/TXP. The results indicate that, when a CMT rising edge is triggered, the common-mode monitoring node Vtcm and the PMOS substrate bias Vbmp rise simultaneously, while Itn1 decreases. On the falling edge, Vtcm and the NMOS substrate bias Vbmn decrease cooperatively, accompanied by a reduction in Itp2. Through dynamic bias adjustment, the differential output signal is stably maintained at approximately 1.4 V. Even under severe common-mode slew rates as high as 220 V/ns, the integrity of the OOK high-frequency pulse signal is preserved. The immunity of the circuit is considerably enhanced by this mechanism.

**Figure 15:**
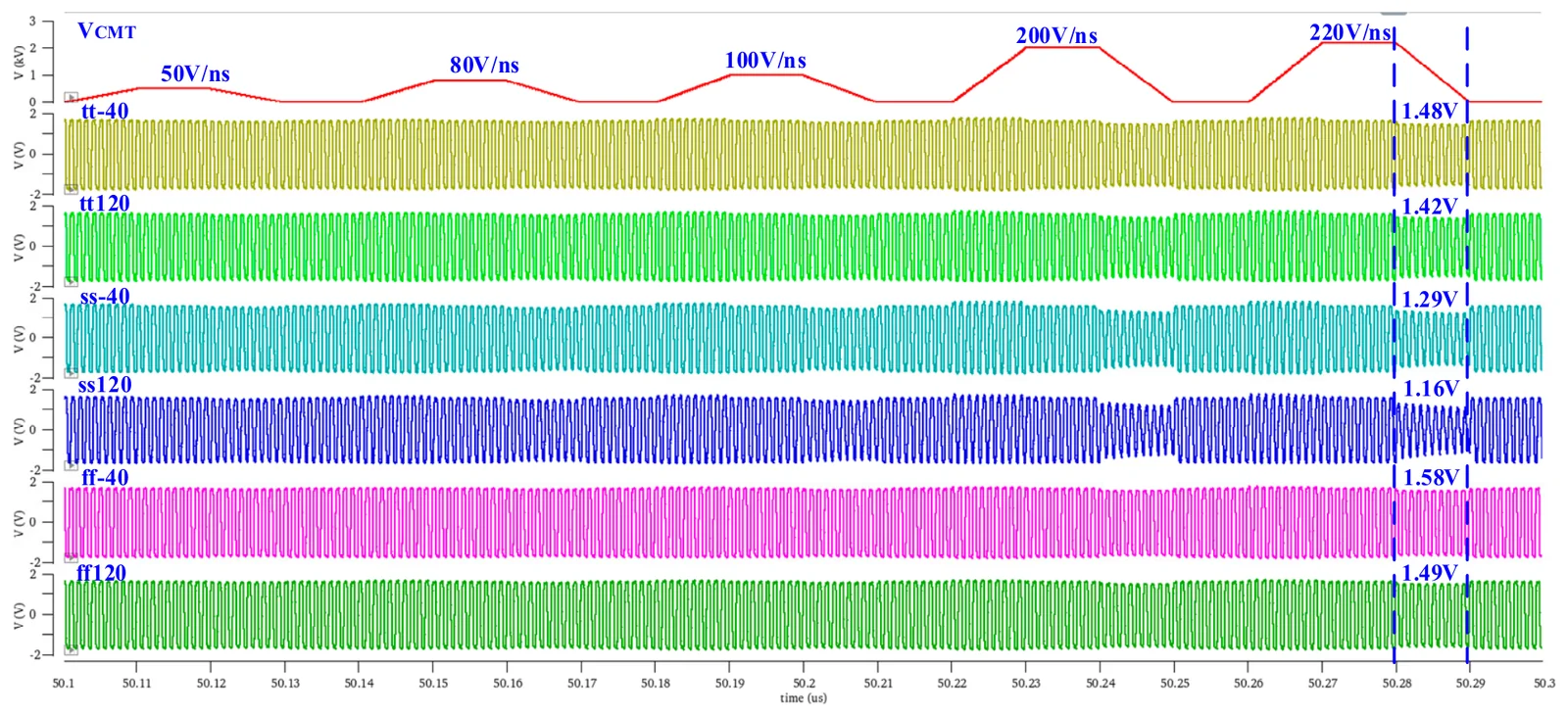
Simulation waveforms of the enhanced OOK architecture under different operating conditions.

To verify the robustness of the proposed common-mode substrate modulation circuit across process corners and temperature range, simulations are performed under common-mode interference slew rates of 50, 80, 100, 200, and 220 V/ns, and the amplitude of the differential output signal Vtxn − Vtxp is observed. The process corners are TT, SS, and FF, and the temperatures are −40 °C and 120 °C. The simulation results under different operating conditions are shown in Figure 15. When the interference slew rate increases to 220 V/ns, the differential output amplitude exhibits a significant degradation. The worst case occurs at the SS corner and 120 °C, where the amplitude drops to 1.16 V. At the TT corner, the amplitudes are 1.48 V and 1.42 V at −40 °C and 120 °C, respectively. At the FF corner, the amplitude is 1.58 V at −40 °C and 1.49 V at 120 °C. Although the amplitude decreases, it remains above VDD/2 (0.9 V) under all conditions, ensuring that the decoding circuit on the receiver side can still reliably identify the signal without bit errors. The results demonstrate that the designed circuit maintains good common-mode transient immunity even under extreme process and temperature conditions, satisfying the system design margin.

**Figure 16:**
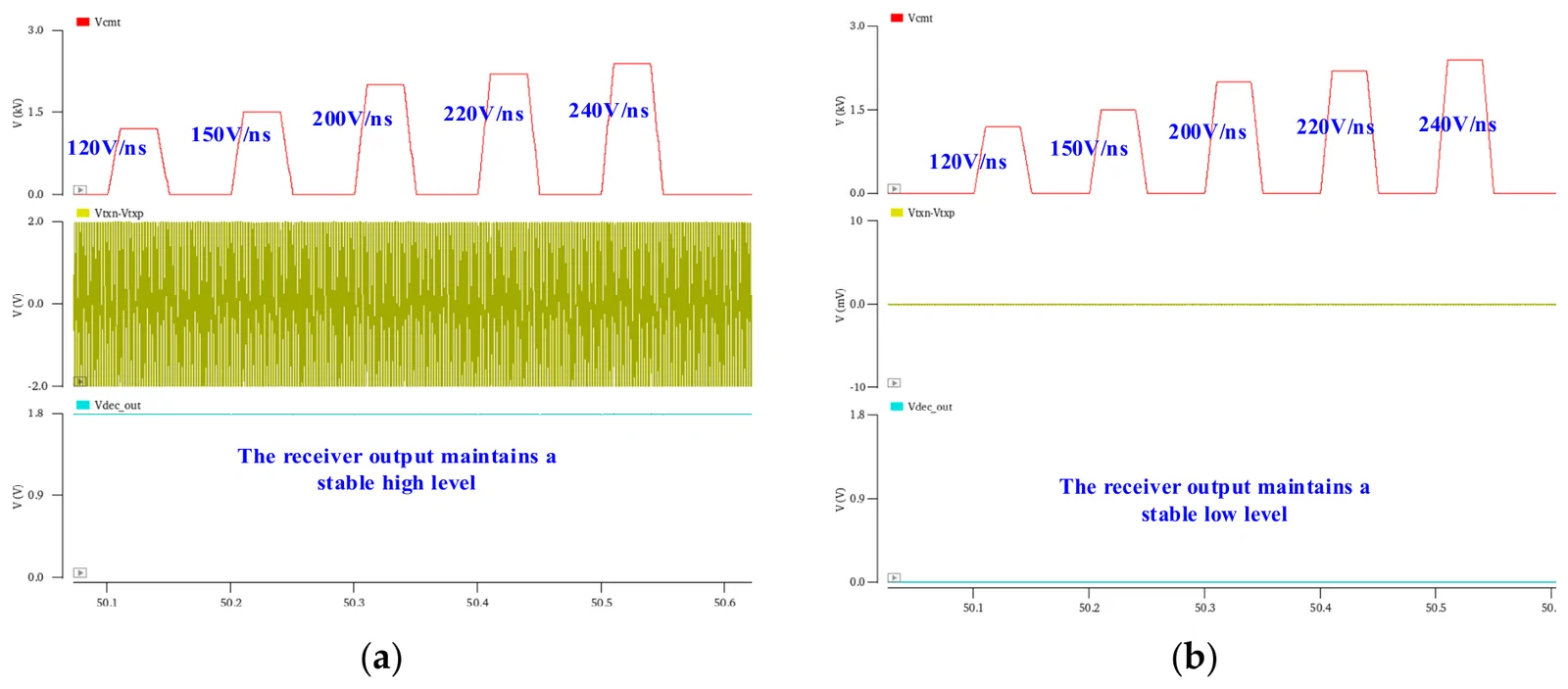
Simulated CMTI performance waveforms. (**a**) VIN is held at logic “1”. (**b**) VIN is held at logic “0”.

To verify the CMTI performance of the proposed system, simulations are performed, as shown in Figure 1. The CMTI capability is evaluated by monitoring the transmission link from the control signal VIN input to the decoded output Vdec_out at the receiver side, observing whether decoding errors occur under common-mode transient (CMT) interference. Figure 16 presents the simulated waveforms when VIN is held at logic “1” and logic “0” under the TT process corner at 25 °C. The results demonstrate that Vdec_out maintains correct decoding even when the CMT interference slew rate is as high as 240 V/ns. Table 3 summarizes the CMTI metrics of the system across different process corners and temperatures, with a minimum achieved CMTI of 220 V/ns. This confirms that the system operates correctly under various process and temperature conditions, verifying its robustness.

## 4. Test Results

### 4.1. Implementation of the Capacitively Isolated Gate Driver Chip

**Figure 17:**
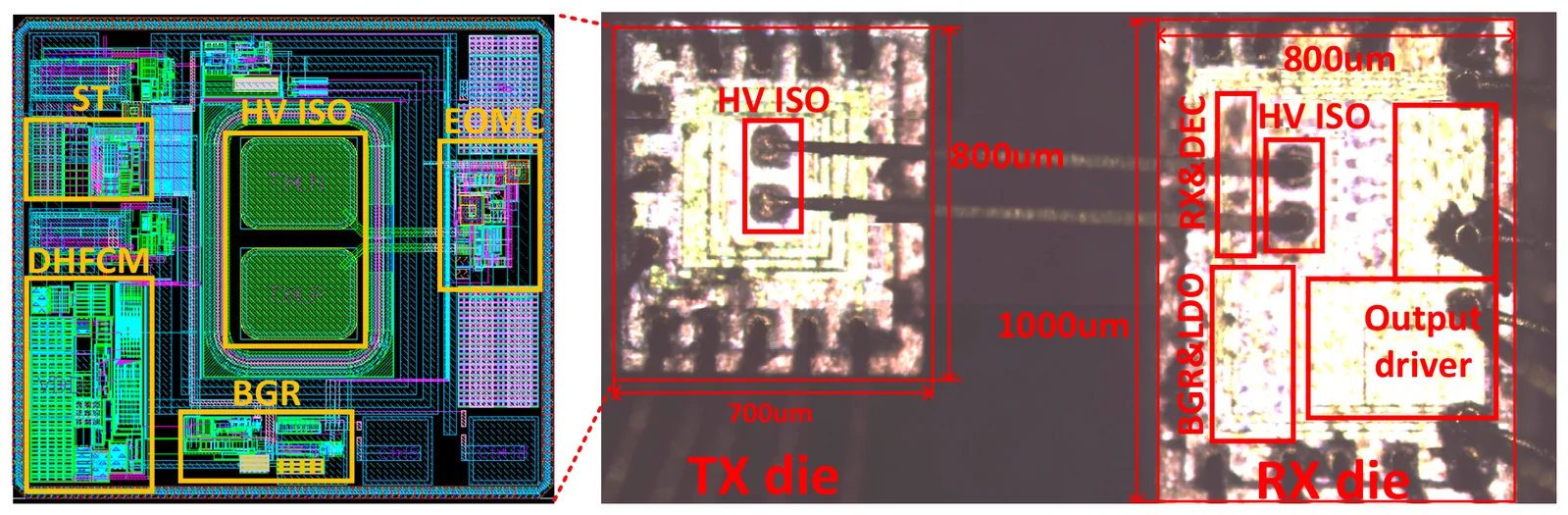
The TX-side layout and chip micrograph of the gate driver IC [28,29].

Figure 17 presents the overall architecture and the TX-side layout of the proposed gate driver chip based on OOK modulation and demodulation, with isolation technology achieving high-voltage isolation. On the TX die, the functional modules are arranged as follows. The ST block is placed in the upper-left corner, which is responsible for shaping the input signal. The DHFCM is located in the lower-left corner. Two HV ISO are integrated in the central region. The bandgap reference (BGR) circuit is positioned below these capacitors. The EOMC is placed beneath the capacitors. On the RX die, an operational amplifier and a decoding circuit are arranged in the upper-left region. A bandgap reference and a low-dropout regulator (LDO) are placed in the lower-left region. Two high-voltage isolation capacitors are also placed in the central area. The output driver stage is located on the far right side, which completes the final signal output.

### 4.2. Test Results and Discussion

**Figure 18:**
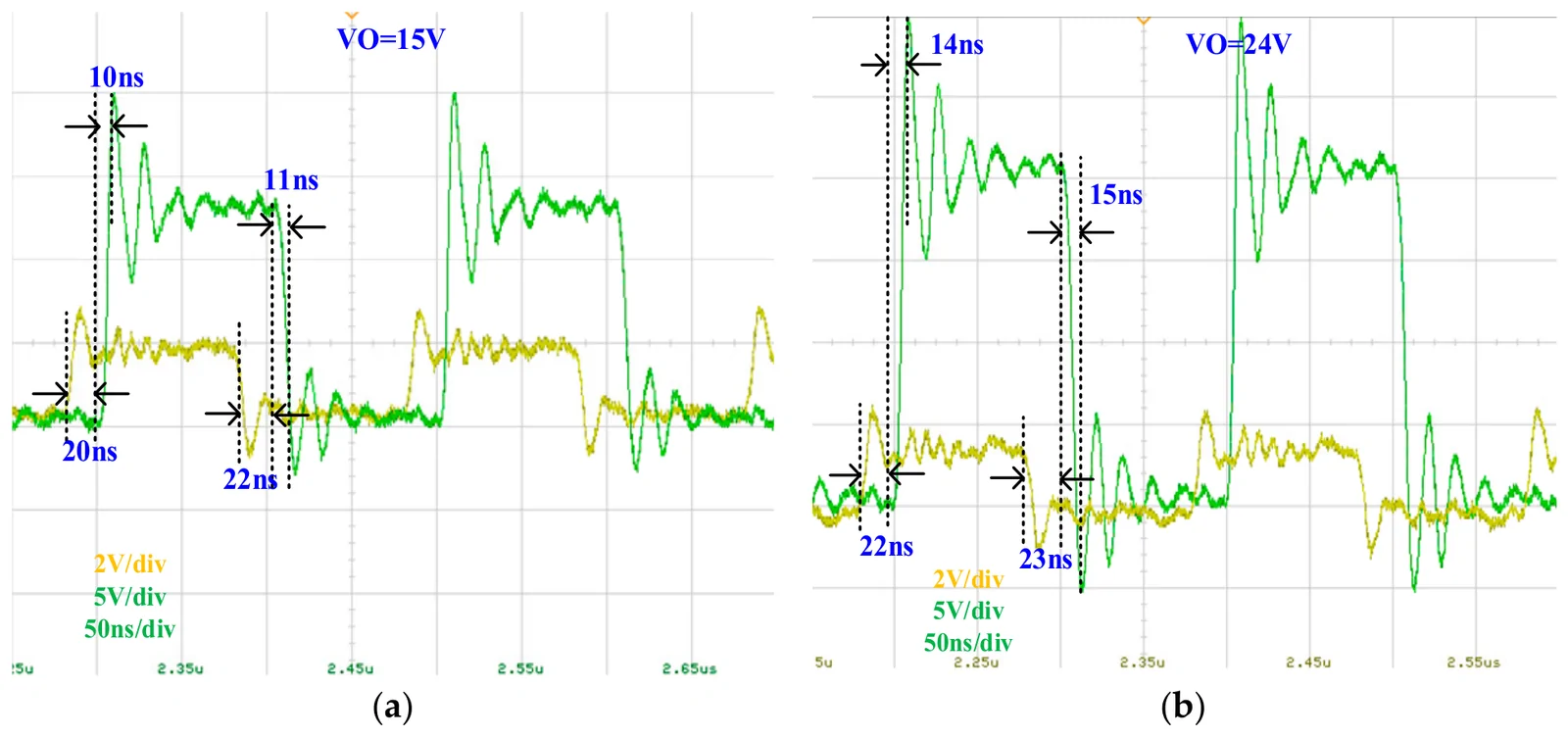
Measured waveforms of gate driver. (**a**) VCC = 15 V @ 5 MHz. (**b**) VCC = 24 V @ 5 MHz.

Figure 18 shows the measured waveforms of the chip under the following test conditions. The input signal frequency is 5 MHz, and the high-side supply voltage VCC of the gate driver chip is set to 15 V and 24 V, corresponding to the recommended minimum and maximum supply voltages, respectively. A 3.3 nF capacitor is employed as the load. When VCC is 15 V, the measured rising-edge and falling-edge propagation delays are 20 ns and 22 ns, respectively. After excluding the intrinsic buffer delay, the corresponding rise time is 10 ns and fall time is 11 ns. Based on these values, the charging current is calculated to be approximately 5.0 A, and the discharging current approximately 4.5 A. When VCC is increased to 24 V, the rising-edge and falling-edge propagation delays are 22 ns and 23 ns, respectively, yielding a rise time of 14 ns and a fall time of 15 ns. The corresponding charging and discharging currents are calculated to be approximately 5.7 A and 5.3 A, respectively. The above results indicate that the driver maintains sufficient driving capability under different supply voltages.

Table 4 summarizes the typical measured results and performance of the proposed driver chip and compares them with those reported in recent literature. Simulated and measured values are distinguished by the labels “Sim.” and “Meas.”

## 5. Conclusions

Based on the OOK modulation architecture, this paper proposes a system-level design strategy that addresses the performance limitations of conventional OOK architectures in both EMI and CMTI. Specifically, a dynamic high-frequency carrier module is employed to reduce EMI radiation, with the suppression effect quantified by carrier spectrum peak reduction, while a dynamic substrate regulation module is introduced to enhance CMTI capability. The chip is fabricated in a 0.18-μm BCD process. Simulation results demonstrate that under different process-temperature corners, the proposed dynamic carrier achieves a spectral peak attenuation of up to 19.621 dB across the fundamental through the third harmonic components, while maintaining approximately 4.43 dB attenuation even under extreme conditions, verifying the robustness of the scheme across all operating corners. Meanwhile, the simulated CMTI reaches 220 V/ns. Chip measurement results show an output voltage range of 15–24 V at a switching frequency of 5 MHz, satisfying the requirements for high-voltage and high-frequency applications. In summary, the proposed strategy effectively breaks the conventional trade-off between carrier spectrum peak reduction (for EMI mitigation) and CMTI enhancement, offering a highly integrated and robust isolated gate-driving solution for high-frequency SiC power systems.

## Figures and Tables

**Table 1:** Simulation results of Vdcs robustness across process corners and temperatures.

Condition	fmax	fmin	fc	Δf	δ
tt (−25)	1.1057	1.0163	1.0610	0.0894	0.084260132
tt (25)	0.9982	0.8956	0.9469	0.1026	0.108353575
tt (100)	0.8767	0.7649	0.8208	0.1118	0.136208577
ss (−25)	0.8620	0.8258	0.8439	0.0362	0.042896078
ss (25)	0.7833	0.7292	0.75625	0.0541	0.071537190
ss (100)	0.7019	0.6259	0.6639	0.0760	0.114475072
ff (−25)	1.3550	1.2054	1.2802	0.1496	0.116856741
ff (25)	1.2154	1.0593	1.13735	0.1561	0.137248868
ff (100)	1.0546	0.8987	0.97665	0.1559	0.159627297

**Table 2:** Simulated FFT spectral peak comparison between the dynamic high-frequency carrier and the fixed-frequency carrier across process corners and temperatures.

Condition	Fundamental Frequency		Second Harmonic		Third Harmonic	
	Fixed Carrier	Dynamic Carrier	Fixed Carrier	Dynamic Carrier	Fixed Carrier	Dynamic Carrier
tt (−25)	−21.8455	−26.284	−47.584	−52.155	−36.869	−41.502
tt (25)	−18.916	−24.341	−44.419	−50.204	−33.668	−38.413
tt (100)	−8.395	−23.561	−34.924	−53.049	−18.261	−37.882
ss (−25)	−23.941	−28.796	−52.257	−57.976	−38.812	−43.632
ss (25)	−21.561	−27.269	−52.095	−58.581	−36.363	−41.995
ss (100)	−16.356	−25.385	−49.276	−58.483	−31.104	−39.514
ff (−25)	−17.308	−22.686	−40.285	−45.683	−31.234	−37.269
ff (25)	−8.711	−24.079	−32.941	−49.451	−22.803	−37.691
ff (100)	−7.921	−21.489	−33.512	−50.418	−18.871	−37.914

**Table 3:** CMTI performance across different process corners and temperatures.

Condition	CMTI (V/ns)
tt (−40)	240
tt (120)	240
ss (−40)	220
ss (120)	230
ff (−40)	240
ff (120)	240

**Table 4:** Performance comparison tables.

**Publication**	[13]	[17]	[21]	[23]	This work
**Isolation**	Cap	Cap	Cap	Transform	Cap
**Modulation**	OOK	Pulse	OOK	Pulse	OOK
**Output voltage (V)**	5 (Sim.)	5 (Meas.)	20 (Sim.)	5 (Sim.)	15~24 (Meas.)
**PWD (ns)**	7.4 (Sim.)	-	-	-	-
**Tdelay (ns)**	50 (Sim.)	100 (Meas.)	15 (Sim.)	5.8 (Sim.)	-
**CMTI (V/ns)**	200 (Sim.)	200 (Meas.)	150 (Sim.)	200 (Sim.)	220 (Sim.)
**Simulated carrier spectrum peak reduction (dB)**	-	-	-	--	4.43 (Sim.)

## Data Availability

All the data are reported/cited in the paper.

## References

[1] Taha W., Nahid-Mobarakeh B., Bauman J. Efficiency Evaluation of 2L and 3L SiC-Based Traction Inverters for 400V and 800V Electric Vehicle Powertrains. Proceedings of the 2021 IEEE Transportation Electrification Conference & Expo (ITEC).

[2] Hirao T., Onishi M., Yasuda Y., Namba A., Nakatsu K. EV Traction Inverter Employing Double-Sided Direct-Cooling Technology with SiC Power Device. Proceedings of the 2018 International Power Electronics Conference (IPEC-Niigata 2018-ECCE Asia).

[3] Roccaforte F., Fiorenza P., Greco G., Lo Nigro R., Giannazzo F., Iucolano F., Saggio M. (2018). Emerging trends in wide band gap semiconductors (SiC and GaN) technology for power devices. Microelectron. Eng..

[4] Simiyu P., Davidson I.E. (2021). MVDC Railway Traction Power Systems; State-of-the Art, Opportunities, and Challenges. Energies.

[5] Adamowicz M., Szewczyk J. (2020). SiC-Based Power Electronic Traction Transformer (PETT) for 3 kV DC Rail Traction. Energies.

[6] Ma Z., Diao F., Guo F., Cao H., Wang Z., Lin N., Wu Y., Mahmud M.H., Zhao Y. (2024). Design and Demonstration of a Medium-Voltage High-Power All Silicon Carbide ANPC Converter with Optimized Busbar Architecture. IEEE J. Emerg. Sel. Top. Power Electron..

[7] Kumar A., Bhattacharya S., Baliga J., Veliadis V. Performance Comparison and Demonstration of 3-L Voltage Source Inverters Using 3.3 kV SiC MOSFETs for 2.3 kV High Speed Induction Motor Drive Applications. Proceedings of the 2021 IEEE Applied Power Electronics Conference and Exposition (APEC).

[8] Biela J., Schweizer M., Waffler S., Kolar J.W. (2011). SiC versus Si—Evaluation of Potentials for Performance Improvement of Inverter and DC–DC Converter Systems by SiC Power Semiconductors. IEEE Trans. Ind. Electron..

[9] Ji S., Zhang L., Huang X., Palmer J., Wang F., Tolbert L.M. (2020). A Novel Voltage Balancing Control with dv/dt Reduction for 10-kV SiC MOSFET-Based Medium Voltage Modular Multilevel Converter. IEEE Trans. Power Electron..

[10] Wang R., Dujic D. (2025). Advanced Dual-Channel Gate Driver with Short-Circuit Protection for Series-Connected Medium-Voltage SiC MOSFETs. IEEE Trans. Power Electron..

[11] Shi G., Yan R., Xi J., He L., Ding W., Pan W., Liu Z., Yang F., Chen D. A Compact 6 ns Propagation Delay 200 Mbps 100 kV/μs CMR Capacitively Coupled Direction Configurable 4-Channel Digital Isolator in Standard CMOS. Proceedings of the 2018 25th IEEE International Conference on Electronics, Circuits and Systems (ICECS).

[12] Angerer M., Schwanninger R., März M. (2020). Active common-mode filter for capacitive-coupled isolated signalling. Electron. Lett..

[13] Zhang X., Liu T., Quan Y., Huang X., Cheng X., Yu Y. (2022). A dV/dt noise canceling circuit of capacitive-isolated gate drivers. IEICE Electron. Express.

[14] Zhao D., Wang Y., Chen Y., Shao J., Wang L., Shao Y., Hua K., Li H., Zhou J., Zeng J. CMTI Improvement Circuit for Digital Isolator. Proceedings of the 2022 IEEE 5th International Electrical and Energy Conference (CIEEC).

[15] Li X., Chen Z., Chu Z., Zhang T., Sun Y., Zhang Y., Zhang Y. CMTI Improvement Circuit for SiC MOSFET Isolated Gate Driver. Proceedings of the 2023 4th International Conference on Advanced Electrical and Energy Systems (AEES).

[16] Zeng J.B., Peng Y.F., Yang Y.H., Xi J.X., Wang T., He L.N. (2023). A capacitively coupled digital isolator with CMTI of 160 kV/μs and data rate of 230 Mbps. Microelectron. J..

[17] Zeng J., Li A., Xi J., He L. (2023). A capacitively coupled digital isolator using multiple-pulse-coding architecture with CMTI of 200kV/μs and static current of 60μA. IEICE Electron. Express.

[18] Li P., Ding Y., Lu C., Song L., Yu X., Qu W. A Capacitively Coupled 3-Channel Digital Isolator with 160 kV/μs Common Mode Transient Immunity. Proceedings of the 2024 International Conference on Energy and Electrical Engineering (EEE).

[19] Pan D., Xiong Z., Lu Q., Miao F., Wu L., Cheng L. (2024). A 250-Mb/s On-Chip Capacitive Digital Isolator with Adaptive Frequency Control. IEEE Solid-State Circuits Lett..

[20] Miao F., Pan D., Xiong Z., Cheng L. A 250-Mbps 2.6-ns Propagation Delay Capacitive Digital Isolator with Adaptive Frequency Control. Proceedings of the 2022 IEEE 65th International Midwest Symposium on Circuits and Systems (MWSCAS).

[21] Zhao Y., Wang L., Chen Z., Li B., Zheng R., Chen C. (2024). A 25 Mbps 15 ns Propagation Delay 150 kV/μs CMTI Configurable Dual-Channel Capacitive Digital Isolation Driver. Micromachines.

[22] Altoobaji I., Hassan A., Ali M., Audet Y., Lakhssassi A. (2024). A Low-Power 0.68-Gbps Data Communication System for Capacitive Digital Isolator with 1.9-ns Propagation Delay. IEEE Trans. Very Large Scale Integr. (VLSI) Syst..

[23] Shao Y., Liu F., Xie Y., Shen M., Li D., Liang Y., Chi B., Yu S. Low-Delay High-CMTI Digital Isolator Based on Set-Reset Modulation Technique Used in IGBT Drivers. Proceedings of the 2024 4th International Conference on Neural Networks, Information and Communication Engineering (NNICE).

[24] Wei Y.J., Lee S.C., Tseng N.H., Yang Y.C., Zeng S.J., Chen K.H., Zhu X., Lin Y.H., Lin S.R., Tsai T.Y. (2025). An Improved Common-Mode Transient Immunity Isolated Gate Driver with On-the-Fly Deadtime Control. IEEE Trans. Circuits Syst. I Regul. Pap..

[25] Hung S.H., Wang T.W., Li S.Y., Hung W.C., Hsu Y.T., Chen K.H., Zheng K.L., Lin Y.H., Lin S.R., Tsai T.Y. (2024). A High Common-Mode Transient Immunity GaN-on-SOI Gate Driver with Quad-Drive Control Technique for High dV/dt 1700-V SiC Power Switch. IEEE J. Solid-State Circuits.

[26] Ding Y., Zhu J., Xu J., Li H., Wang S., Li H., Yan C., Liu W. (2026). A 250-Mb/s On-Chip Capacitive Digital Isolator with 200 kV/μs Common-Mode Transient Immunity. IEEE Trans. Very Large Scale Integr. (VLSI) Syst..

[27] Dong Y., Wu R. A High-CMTI Receiver Front-End Circuit for Capacitive-Coupled Digital Isolators. Proceedings of the 2026 5th International Conference on Electronics, Integrated Circuits and Communication Technology (EICCT).

[28] Li J., Lu J., Tie R., Yang D., Wu Z., Chen Z., Jin C., Yu Z., Gu X., Yin Y. (2026). A capacitively coupled SiC MOSFET gate driver using dv/dt enhanced OOK transmitter with CMTI of 220 V/ns. IEICE Electron. Express.

[29] Li J., Lu J., Tie R., Yang D., Wu Z., Chen Z., Jin C., Yu Z., Gu X., Yin Y. (2026). A 5 A 10 MHz capacitively coupled SiC MOSFET gate driver with 220 V/ns CMTI and 15 to 30 V Output Drive Voltage. Microelectron. J..

[30] Yang C., Chen W., Fan Y., Gui P. (2021). Design and Characterization of a 10-MHz GaN Gate Driver Using On-Chip Feed-Forward Gaussian Switching Regulation for EMI Reduction. IEEE J. Solid-State Circuits.

[31] Tsai H.Y., Zeng S.J., Liang Y.T., Chen K.H., Zheng K.L., Li C.C. A Monolithic Low-ILEAK Cross-Coupled GaN Driver with ΔΦ-Reduced EMI-Rejecter for 21.51dBµV-EMI-Reduction and 1/10x filter-capacitor. Proceedings of the 2024 IEEE Symposium on VLSI Technology and Circuits (VLSI Technology and Circuits).

[32] Han D., Kim S., Dong X., Guo Z., Li H., Moon J., Li Y., Peng F.Z. An Integrated Active Gate Driver for Half-bridge SiC MOSFET Power Modules. Proceedings of the 2022 IEEE Applied Power Electronics Conference and Exposition (APEC).

[33] Guo Z., Li H., Cheetham P. A Very-High-Frequency Isolated Gate Driver Power Supply Using Solid Dielectrics for Medium Voltage SiC MOSFETs. Proceedings of the 2022 IEEE Applied Power Electronics Conference and Exposition (APEC).

[34] Ding Y., Lu C., Song L., Yu X., Qu W. A Fully integrated, Low-Cost, High Channel Utilization Digital Isolator in Standard CMOS with 50Mbps and 100kV/μs CMTI. Proceedings of the 2024 9th Asia Conference on Power and Electrical Engineering (ACPEE).

[35] Kwak J.W., Ma D.B. An Automotive-Use Dual-fsw-Zone Hybrid Switching Power Converter with Vo-Jitter-Immune Spread-Spectrum Modulation. Proceedings of the ESSCIRC 2022—IEEE 48th European Solid State Circuits Conference (ESSCIRC).

[36] Mao Y., Gao H., Shao J., Lin X., Wu P., Lu P., Xu H., Yan N. (2025). An All-Digital Spread-Spectrum Clock Generator with Feedforward Gain Calibration for LPWAN Chirp Transmission System. IEEE Trans. Circuits Syst. II Express Briefs.

[37] Zhang Y., Cao Z., Zeng Y., Meng X., Yin Y. A Low-Power Open-Loop Spread-Spectrum Clock Generator Design for Ref-Clk Applications. Proceedings of the 2025 IEEE Asia Pacific Conference on Circuits and Systems (APCCAS).

[38] Sun Z., Li Y., Wang H., Guo M., Zhang X. A Hybrid Modulation Strategy for SiC Inverters Integrating Secondary Frequency Modulation and Double-Modulation-Wave Technique. Proceedings of the 2026 IEEE 9th International Electrical and Energy Conference (CIEEC).

[39] Yang C.Y., Wang S.C., Yu W.Y., Shih M.S., Lin P.T. (2026). A 2.9-Gb/s Spread-Spectrum Clocking Transceiver Utilizing Phase Interpolation for EMI-Suppressed Chip-to-Chip Links. IEEE Trans. Circuits Syst. I Regul. Pap..

[40] Tsou M.C., Chen Q.M. (2026). Valley-Preserving Spread-Spectrum Jitter for Quasi-Resonant Flyback Converters to Reduce Conducted EMI. IEEE Open J. Power Electron..

[41] Zhong R., Li Z., Song Y. Design and Implementation of High-Performance and Low-Power Spread-Spectrum Clock Phase-Locked Loop Based on Ring Oscillator. Proceedings of the 2026 11th International Conference on Computer and Communication Systems (ICCCS).

[42] Lin J., Zhang J., Shao S., Chen Z. (2026). Power Fluctuation Suppression of Quasi-Resonant Flyback Converter with Frequency Jittering. IEEE Trans. Power Electron..

